# Blockade of Angiotensin II Receptor Type 1 Abolishes the Erythropoietin Response to Exercise

**DOI:** 10.1093/function/zqaf032

**Published:** 2025-07-21

**Authors:** Meihan Guo, David Montero

**Affiliations:** Department of Medicine, Beth Israel Deaconess Medical Center, Harvard Medical School, Boston, MA 02115, USA; Faculty of Medicine, School of Public Health, Hong Kong University, Pokfulam, Hong Kong; Faculty of Medicine, School of Public Health, Hong Kong University, Pokfulam, Hong Kong; Department of Medicine, School of Clinical Medicine, Hong Kong University, Pokfulam, Hong Kong; Libin Cardiovascular Institute of Alberta, University of Calgary, Calgary, Alberta T2N 1N4, Canada

**Keywords:** erythropoietin, exercise-induced erythropoiesis, antihypertensive medication, renin-angiotensin system, angiotensin II type 1 receptor, randomized controlled trial

## Abstract

Beneficial adaptations to exercise depend on the normal function of the endocrine system. Whether commonly prescribed antihypertensive medication inhibits erythropoietin (EPO) production with exercise, a key response to enhance aerobic capacity, remains unknown. Healthy adults (*n* = 63, 42.3 ± 16.5 yr, 52% ♀) matched by age, sex, and physical activity were randomized in a blinded and crossover manner to orally ingest valsartan (angiotensin II type 1 receptor-blockade, AT1-blockade) or placebo (calcium carbonate, PBO) 4 h before starting the experimental protocol. Before and after 1 h of moderate cycling exercise, blood samples were taken to measure circulating EPO and EPO-regulating hormones along with blood pressure. Cardiac structure/function and peak pulmonary O_2_ consumption (VO_2peak_) were assessed during exercise. AT1-blockade decreased heart volumes (left atrium and ventricle) during exercise compared with PBO, particularly in men (*P* ≤ 0.036). Whole-body O_2_ extraction and VO_2peak_ were unaffected by AT1-blockade irrespective of sex (*P* ≥ 0.325). Before and after exercise, AT1-blockade reduced arterial blood pressures (systolic, diastolic) in both sexes (*P* < 0.001). A condition × time interaction was detected for circulating EPO (*P* = 0.002), such that AT1-blockade decreased EPO at 3-h post-exercise compared with PBO (*P* ≤ 0.025). The effect of exercise on EPO-regulating hormones (angiotensin II, aldosterone, copeptin) was diminished with AT1-blockade. Sex *per se* did not influence the endocrine response to AT1-blockade. In conclusion, in a randomized, double-blind and placebo-controlled study design, AT1-blockade abolishes the acute EPO response to exercise in women and men. Antihypertensive medications hindering AT1 signaling may restrict key endocrine responses to exercise.

## Introduction

The exercise-induced increase in circulating erythropoietin (EPO), ie, the hormone that regulates red blood cell production, is consider crucial for the enhancement of peak O_2_ consumption (VO_2peak_), the hallmark of aerobic exercise capacity.^1–3^ Accordingly, a pharmacological intervention that hinders this erythropoiesis-stimulating mechanism may alter one of the strongest predictors of cardiovascular and all-cause mortality in the general population.^4^

Among ˜700 million adults diagnosed with hypertension worldwide, approximately 75% of them receive antihypertensive medication.^5^ The most common class of antihypertensive medication in the US population comprises angiotensin converting enzyme inhibitors (ACEi) and angiotensin receptor blockers (ARB), with a prevalence ≥66%.^6^ Hence, at least 129 million individuals in the United States are taking these medications. ACEi and ARB block the renin-angiotensin system (RAS), thus attenuating the vasoconstrictor effect of angiotensin II (ANGII). Yet, additional effects of ANGII include the stimulation of EPO production in kidney cells via the activation of early growth response 1 protein and the p21ras-extracellular signal-regulated kinase 1/2 axis, mediated by the angiotensin II receptor type 1 (AT1).^7^ This EPO-stimulating effect is blocked by ACEi and ARB. Indeed, chronic treatment with ACEi and/or ARB leads to a ∼20-60% reduction in basal circulating EPO,^8–10^ leading to true anemia.^8^ Likewise, these antihypertensive drugs became the mainstay of treatment for excessive erythropoiesis in kidney transplant patients three decades ago.^11^ The question that remains unanswered is the following: do the most prevalent prescribed class of antihypertensive medication inhibit the EPO response to exercise (which could also be elicited by RAS-independent mechanisms)?^3^ If so, the pharmacological control of hypertension in most individuals would have a high price: the curtailment of substantial improvements in aerobic exercise capacity and its associated major health benefits.

Using a randomized, double-blind, and placebo-controlled design, this study sought to determine whether a commonly used antihypertensive medication, which blocks the receptor of ANGII that stimulates erythropoiesis (AT1-blockade), inhibits the acute EPO response to exercise in healthy women and men throughout the adult lifespan. An ARB, specifically valsartan, was chosen as the experimental drug owing to: (1) more specific blockade (downstream the RAS signaling cascade) than ACEi; (2) known antihypertensive effect and pharmacokinetics of a single dose (80 mg) in healthy adults,^12^ including our study population;^13^ (3) peak plasma concentration, endocrine and hypotensive effects are observed at 3-4 h, then remaining approximately stable for the next ∼4 h,^12,13^ concurring in time with the expected acute response of EPO to exercise in healthy adults.^2^ This study additionally characterized the effect of AT1-blockade on exercise-induced effects on EPO-regulating hormones as well as cardiac, hemodynamic, O_2_ consumption and extraction variables during incremental exercise, which might influence the EPO response to exercise.

## Methods

### Ethical Approval and Clinical Trial Registration

This study, a randomized controlled trial (RCT), was approved by the Institutional Review Board of the University of Hong Kong/Hospital Authority West Cluster (UW 22-025) and conducted in accordance with the declaration of Helsinki. Prior to the start of the experiments, informed oral and written consents were obtained from the participants. The RCT was registered at ClinicalTrials.gov (NCT05269615) on April 2022.

### Study Participants

Healthy women [*n* = 33, 42.0 ± 16.6 (18.4-66.2) yr] and men [*n* = 30, 42.7 ± 16.4 (21.8-68.7) yr] matched by age and physical activity were recruited via printed/online advertisements in the city of Hong Kong. Exercise training history and moderate-to-vigorous physical activity (MVPA, total and specific to endurance exercise) over participant’s lifetime and thoroughly detailed during the last three months prior to the study were determined at screening, as previously described.^14^ Inclusion criteria comprised healthy status according to health/clinical questionnaires and resting echocardiography/ECG screening, absence of current medical symptoms and medication, and no history of disease.

### Study Design

Participants were required to report twice to the laboratory for testing. The experimental protocol in both sessions was identical except for the pill taken before testing. Specifically, 4 h before starting the measurements, the participants orally ingested valsartan (80 mg, Novartis Pharmaceuticals) for AT1-blockade, or calcium carbonate (500 mg, Shandong Yuwang Pharmaceutical) for placebo (PBO). The timing of pill ingestion was planned to induce peak hemodynamic and endocrine effects of AT1-blockade within the measurement period of each study visit (from the fourth to the seventh hour after pill ingestion).^12,13^ The ideal PBO (ie, a valsartan pill without the active component but identical in shape, size, color, and taste) was not obtained despite multiple requests to the company (Novartis Pharmaceuticals) were made from our research group. Nonetheless, at the end of the second visit, no participant was confident to conjecture the type of pill (BP-lowering or PBO) they took in each visit, therefore they were considered as blinded. Likewise, all investigators except one (M.G.) were blinded to the pill allocation. The pill taken in the first visit (and thus the remaining pill taken in the second visit) was sequentially determined for each participant via covariate adaptive randomization, with sex, age, and physical activity as covariate categories. Time of day for the start of testing sessions was invariable for each participant (a specific time within the 2:00-4:00 pm range) and matched between conditions (AT1-blockade, PBO) and sex groups, with 2-7 d between the first and second sessions.

The participants were instructed to avoid strenuous exercise, alcohol and caffeine 24 h prior to testing. All measurements were performed after a fasting period (≥4 h) to avoid postprandial hemodynamic alterations.^15^ According to previous studies, the menstrual phase was noted but not fixed for testing as it does not modify the study outcomes.^16,17^ Prior to starting the experimental protocol, the participants completed health and clinical questionnaires. Thereafter, the participants were subjected to anthropometric and body composition assessments, and rested in supine position for 15 min on the testing platform in order to stabilize hematological, cardiovascular and hemodynamic variables. During this resting period, the most prominent vein in the antecubital fossa of the left arm was cannulated (Introcan Safety^®^ IV Catheter, 22 G, B. Braun Medical) for blood sampling throughout the visit. Once the initial blood sample (4 mL) was taken and the cardiac and hemodynamic measurements were completed at rest, the incremental exercise test started, lasting 7-9 min and comprising continuous cardiac, hemodynamic and pulmonary gas exchange measurements, as subsequently described in the “Measurements” section. Then, the participants rested for 5 min before performing 1 h of cycle ergometry (Monark 828 E) at moderate intensity: 80% of the peak heart rate (HR_peak_) obtained in the precedent incremental exercise test. Immediately after finishing the cycle ergometry, the participants were placed on a comfortable upright chair with a desk for a 3-h resting period. Blood samples (4 mL per sample) were taken and central hemodynamics (CNAP^®^ Monitor 500 HD, CNSystems) measured at 0-, 1-, 2-, and 3-h time points during this final period (post-exercise). Due to the ∼3-h time period required to observe exercise-induced changes in circulating EPO,^2^ post-exercise measurements for EPO were not performed at intermediate points (0-h, 1-h, 2-h) post-exercise). The participants were allowed to drink water ad libitum in a volume-graded bottle during the continuous cycle ergometry exercise and subsequent 3-h resting period; the amount of water ingested was recorded and considered as a potential covariate.

### Measurements

Body composition. Body composition was assessed via dual-energy x-ray absorptiometry (DXA) (Hologic QDR 4500; Hologic), as previously described.^18–20^

Cardiac structure, function, and hemodynamics. Apical 4-chamber and 2-chamber cine-loops were recorded and assessed offline via high-resolution ultrasound (M9, Mindray Medical; TOMTEC, Philips) at rest and during progressively increasing levels of exercise intensity (60, 70, 80, 90, and 100% HR_peak_) during the incremental exercise test (detailed in the following subsection). According to the American Society of Echocardiography and the European Association of Cardiovascular Imaging recommendations, cardiac chamber quantification including left ventricular (LV) end-diastolic and end-systolic volumes (LVEDV, LVESV) was performed using the modified Simpson method (biplane method of disks) by tracing the endocardial border of the LV in apical 4-chamber and 2-chamber views at end-diastole and end-systole.^21,22^ The difference between LVEDV and LVESV provided stroke volume (LV SV). Cardiac output (LV Q) was calculated as the product of LV SV and heart rate (HR). Right (RA) and left atrial (LA) volumes were determined from the apical 4-chamber view at end-systole, right before mitral valve opening. Cardiac volumetric variables are commonly normalized by body surface area (BSA = 0.007184 × weight^0.425^ × height^0.725^) to eliminate the confounding impact of body size and thus were accordingly presented.^23^ Regarding the assessment of LV function, transmitral inflow velocities were determined by pulsed-wave Doppler, with the sample volume placed between the mitral leaflet tips in the apical 4-chamber view throughout incremental exercise. The peak inflow velocities during early (E) and late (A) diastole were measured, and the E/A velocity ratio was calculated. The assessment of LV function also included myocardial tissue e’ and a’ velocities measured via tissue Doppler imaging in the septal ventricular wall adjacent to the mitral annulus.

Arterial blood pressures [systolic blood pressure (SBP), diastolic blood pressure (DBP) and mean arterial pressure (MAP)] were continuously measured at baseline rest, during incremental exercise and for the last 10 min of each hour of the final resting period via the volume-clamp method in the middle finger of the left hand, calibrated to brachial artery pressure and positioned at the heart height level (CNAP^®^ Monitor 500 HD, CNSystems). Total peripheral resistance (TPR) was calculated by the ratio of MAP and LV Q. Circulating hemoglobin mass was measured via the carbon monoxide rebreathing technique,^18^ and plasma volume was determined with the additional measurement of hemoglobin concentration and hematocrit (ABL80, Radiometer) from venous blood samples taken at baseline rest and 3-h post-exercise.

Aerobic capacity. Pulmonary O_2_ uptake (VO_2_), CO_2_ output and ventilation were recorded via a mixing chamber system (KORR Medical) throughout the established incremental cycle ergometry exercise test in our laboratory.^18^ Following a warm-up period at 10-30 W, the workload was progressively increased by 10-30 W increments every 50 s until exhaustion was reached in the recommended total duration of 7-9 min.^24^ Calibration of the gas analyzers and the flowmeter was conducted prior to each test. Values were averaged over 15 s following current recommendations.^25^ The highest average value defined VO_2peak_ provided that at least two of the following established criteria were fulfilled: (i) plateau in O_2_ uptake despite increased workload, (ii) age- and body position-predicted HR_peak_ ± 10 bpm^26,27^ and/or (iii) RER > 1.^28^ The arterio-venous O_2_ difference (a-vO_2diff_) during incremental exercise was determined by the Fick Principle (VO_2_ = LV Q × a-vO_2 diff_).

Hormones. Venous blood samples (5 mL) were collected in EDTA tubes at 3 time points: baseline rest, 0-h post-exercise, 3-h post exercise. Before blood collection, 50 mL of Pierce Protease Inhibitor solution (Pierce Protease Inhibitor Tablets, Thermo Fisher Scientific) was poured into the EDTA tubes to enhance the stability of RAS hormones, as recently recommended.^29^ The blood samples were collected anaerobically and stored at 4°C after collection and then centrifuged at 1500 × *g* at 4°C for 10 min. The resulting plasma samples were aliquoted into vials and stored at −80°C until further analysis. Plasma EPO (Human EPO ELISA Kit, Thermo Fisher Scientific) and EPO-regulating hormones such as renin (Human Renin ELISA Kit, Thermo Fisher Scientific), ANGII (Human Angiotensin II ELISA Kit, Thermo Fisher Scientific), aldosterone (Competitive Aldosterone ELISA Kit, Thermo Fisher Scientific), copeptin (Human Copeptin ELISA Kit, Thermo Fisher Scientific), and cortisol (Human Cortisol ELISA Kit, Elabscience^®^) were quantified using enzyme-linked immunosorbent assays (ELISA). Standard curves were generated using the manual protocol with either the 4PL or 5PL fitting method as recommended for the ELISA Kit, with R-squared values ranging from 0.995 to 0.95. The intra-assay coefficient of variation (CV%) ranged from 6.3 to 9.6% for all hormones.

### Statistical Analysis

Statistical analyses were performed using SPSS 26.0 (IBM). Data were tested for normal distribution with the Kolmogorov-Smirnov test and for homogeneity of variances with the Levene’s test. The primary outcome was the 3-h EPO response to endurance exercise (1-h cycle ergometry at 80% HR_peak_) following AT1-blockade compared with PBO. According to power analyses of a precedent study in our laboratory assessing the EPO response to 1 h of cycle ergometry (at a similar intensity as in the current study) in healthy women and men,^2^ a sample size of 9 participants provided >85% power to detect a 3.5 mlU mL^−1^ difference in EPO at 3-h post-exercise between experimental conditions (plausibly modifying the renin-angiotensin system). Since no previous study had specifically compared the EPO response to exercise between AT1-blockade and PBO, we largely increased the sample size in this study (*n* ≥ 30) to secure high statistical power. General baseline variables in the PBO visit were compared between sexes via the independent *t*-test. Three-way ANOVA with repeated measures was implemented to assess the effects of the experimental condition (AT1-blockade vs. PBO), sex, and exercise intensity or time during incremental exercise or resting periods, respectively. The interaction between these factors (condition, sex, exercise intensity or time) was additionally determined with 3-way ANOVA, including post-hoc comparisons between conditions and sexes at any time point. A two-tailed *P*-value less than 0.05 was considered significant. All data were reported as mean ± SD unless otherwise stated.

## Results

### Baseline Characteristics

Table 1 presents general characteristics at baseline according to sex. Age and physical activity (MVPA total and endurance-specific) were closely matched between sexes (*P* ≥ 0.556). All individuals were non-smokers and non-obese [body mass index (BMI) < 30 kg m^−2^], with anthropometrical and body composition variables differing between sexes as expected. Aerobic capacity was greater in men compared with women (*P* < 0.001), both sexes falling in the age- and sex-specific 90th percentile of VO_2peak_ in the healthy adult population.^30^ Resting arterial blood pressures were within the normotensive range,^31^ with higher values in men relative to women (*P* ≤ 0.033). Regarding cardiac variables at rest, LV volumes (LVEDV, LVESV, LV SV), with or without normalization by BSA, were larger in men compared with women (*P* < 0.001). Measures of LV function at rest did not differ between sexes (*P* ≥ 0.164).

**Table 1.** General Baseline Characteristics of Study Participants

	♀	♂
*n*	33	30
Age (yr)	42.0 ± 16.6	42.7 ± 16.4
Height (cm)	158.1 ± 5.5	171.7 ± 5.8*
Weight (kg)	55.3 ± 6.5	68.9 ± 10.3*
BMI (kg m^−2^)	22.1 ± 2.5	23.4 ± 3.7
BSA (m^2^)	1.55 ± 0.10	1.81 ± 0.13*
SBP (mm Hg)	103.7 ± 13.5	119.8 ± 15.2*
DBP (mm Hg)	67.7 ± 11.7	74.4 ± 12.4*
MVPA (h wk^−1^)	5.0 ± 3.0	5.4 ± 2.2
MVPA-END (h wk^−1^)	4.3 ± 3.1	4.7 ± 2.1
Smoking (%)	0	0
VO_2peak_ (mL min^−1^ kg^−1^)	28.0 ± 6.1	37.1 ± 8.4*
Body composition		
BMC (kg)	1.85 ± 0.30	2.41 ± 0.37*
LBM (kg)	37.1 ± 3.4	51.4 ± 5.8*
Absolute fat (kg)	17.5 ± 4.6	16.1 ± 6.4*
Relative fat (%)	30.7 ± 5.3	22.5 ± 5.8*

Data are reported as mean ± SD.

Data obtained in the PBO visit are presented.

*P < 0.05 between sexes (men vs. women).

BMC, bone mineral content; BMI, body mass index; BSA, body surface area; DBP, diastolic blood pressure; LBM, lean body mass; MVPA, total moderate-to-vigorous physical activity; MVPA-END, moderate-to-vigorous physical comprising endurance exercise; PBO, placebo; SBP, systolic blood pressure; VO_2peak_, peak O_2_ uptake.

### Effect of Angiotensin II Type 1 Receptor (AT1)-Blockade During Incremental Exercise

Table 2 shows the effect of AT1-blockade on key cardiac, hemodynamic, O_2_ consumption and extraction variables during incremental exercise. AT1-blockade resulted in decreased LA and LV volumes (LVEDV, LV SV) compared with PBO (*P* ≤ 0.036). LV Q was also reduced with AT1-blockade relative to PBO (*P* = 0.029). An interaction was found for LV Q among condition, sex, and exercise intensity (*P* < 0.001), in that AT1-blockade generally reduced LV Q in men, but not in women, throughout incremental exercise. As for LV function, AT1-blockade decreased LV E/e’, a marker of LV filling pressures, compared with PBO (*P* = 0.006). Regarding the effect of sex, men exhibited larger LV volumes and output (with and without normalization by BSA), SBP, VO_2_, and a-vO_2_diff than women (*P* ≤ 0.029). A more comprehensive set of effects on cardiac and hemodynamic variables during incremental exercise is presented in Supplemental Table S1.

**Table 2.** Effect of Angiotensin II Type 1 Receptor (AT1)-Blockade on Key Selected Cardiac, Hemodynamic, O_2_ Consumption, and Extraction Variables During Incremental Exercise

	Exercise intensity (% HR_peak_)					Three-way ANOVA (*P* value)		
	60	70	80	90	100	Condition^a^	Sex	Exercise intensity
**Heart volumes and function**								
LA (mL m^−2^)						**0.036**	0.276	**<0.001**
♀ AT1-blockade	12.8 ± 2.8	12.9 ± 2.3	12.2 ± 2.4	12.0 ± 2.7	11.3 ± 2.3†			
♀ PBO	13.2 ± 3.3	12.8 ± 3.3	12.7 ± 2.9	12.9 ± 2.8	12.4 ± 2.6			
♂ AT1-blockade	13.3 ± 3.4	12.7 ± 3.2†	13.1 ± 3.3	13.4 ± 3.8	12.8 ± 3.7			
♂ PBO	13.8 ± 3.8	13.6 ± 3.6	13.5 ± 3.8	13.3 ± 3.5	13.2 ± 3.3			
LVEDV (mL m^−2^)						**0.017**	**<0.001**	**0.001**
♀ AT1-blockade	61.7 ± 10.5	62.1 ± 10.4	63.8 ± 8.6	61.9 ± 9.5†	64.7 ± 11.5			
♀ PBO	60.5 ± 10.5	62.7 ± 8.9	64.0 ± 9.5	65.0 ± 10.6	63.3 ± 10.3			
♂ AT1-blockade	72.4 ± 11.8*	72.7 ± 11.4*	72.2 ± 11.2†*	74.2 ± 10.9*	72.1 ± 10.0†*			
♂ PBO	73.3 ± 12.2*	74.3 ± 11.1*	76.2 ± 10.9*	76.9 ± 10.4*	77.5 ± 12.0*			
LV SV (mL m^−2^)						**0.005**	**<0.001**	**<0.001**
♀ AT1-blockade	50.5 ± 9.4	51.3 ± 9.1	53.1 ± 7.4	52.1 ± 8.1†	54.9 ± 10.2			
♀ PBO	49.7 ± 9.0	52.0 ± 7.4	53.0 ± 7.8	54.8 ± 9.2	53.5 ± 9.6			
♂ AT1-blockade	58.3 ± 10.0*	59.7 ± 10.0*	58.9 ± 9.6†*	61.1 ± 9.1†*	60.0 ± 8.4†*			
♂ PBO	59.3 ± 10.6*	61.2 ± 10.2*	63.0 ± 9.6*	64.4 ± 9.0*	65.4 ± 11.1*			
LV Q (L min^−1^ m^−2^)						**0.029**	**0.002**	**<0.001**
♀ AT1-blockade	4.64 ± 1.03	5.51 ± 1.16	6.49 ± 1.14	7.13 ± 1.39	8.31 ± 1.90			
♀ PBO	4.55 ± 1.06	5.52 ± 0.99	6.46 ± 1.13	7.52 ± 1.57	8.06 ± 1.72			
♂ AT1-blockade	5.35 ± 1.32*	6.39 ± 1.46*	7.22 ± 1.54*	8.44 ± 1.62*	9.09 ± 1.79			
♂ PBO	5.42 ± 1.18*	6.56 ± 1.38*	7.76 ± 1.53*	8.87 ± 1.71*	9.75 ± 2.08*			
LV E/e'						**0.006**	0.114	**<0.001**
♀ AT1-blockade	5.19 ± 1.15	5.42 ± 1.03	5.98 ± 1.99	5.98 ± 1.27	6.05 ± 1.43			
♀ PBO	5.58 ± 1.35	5.95 ± 1.33	5.87 ± 1.26	6.15 ± 1.66	6.10 ± 1.39			
♂ AT1-blockade	4.91 ± 0.88	4.96 ± 1.21†	5.08 ± 1.47†	5.46 ± 1.99	5.59 ± 1.65			
♂ PBO	5.27 ± 1.21	5.57 ± 1.29	5.92 ± 1.27	6.21 ± 1.46	6.15 ± 1.40			
**LV work, arterial blood pressure, and vascular resistance**								
LV work (mm Hg L min^−1^)						0.054	**<0.001**	**<0.001**
♀ AT1-blockade	663 ± 202	826 ± 241	1020 ± 277	1190 ± 379	1452 ± 573			
♀ PBO	665 ± 173	842 ± 223	1080 ± 298	1322 ± 368	1461 ± 465			
♂ AT1-blockade	932 ± 327*	1183 ± 405*	1394 ± 501*	1738 ± 566*	1906 ± 526*			
♂ PBO	969 ± 312*	1239 ± 404*	1560 ± 487*	1884 ± 564*	2112 ± 677*			
SBP (mm Hg)						0.835	**0.029**	**<0.001**
♀ AT1-blockade	116.2 ± 20.3	116.2 ± 22.1	121.6 ± 23.4	127.1 ± 27.2	128.7 ± 32.8			
♀ PBO	115.6 ± 22.9	118.1 ± 26.3	124.4 ± 27.8	129.0 ± 26.0	131.0 ± 27.9			
♂ AT1-blockade	129.2 ± 19.0*	133.2 ± 22.0*	135.6 ± 27.2	142.9 ± 29.1*	143.1 ± 29.1			
♂ PBO	127.5 ± 22.9*	130.2 ± 25.6*	133.2 ± 27.8	138.7 ± 33.1	139.0 ± 34.0			
TPR (dyn s cm^−5^)						0.726	**<0.001**	**<0.001**
♀ AT1-blockade	1088 ± 2.92	937 ± 2.72	836 ± 223	816 ± 222	712 ± 201			
♀ PBO	1150 ± 331	957 ± 234	866 ± 224	808 ± 248	775 ± 194			
♂ AT1-blockade	851 ± 234*	745 ± 218*	691 ± 197*	627 ± 163*	614 ± 190			
♂ PBO	854 ± 212*	741 ± 156*	654 ± 136*	614 ± 155*	573 ± 155*			
**Whole-body O_2_ uptake and extraction**								
VO_2_ (mL min^−1^ kg^−1^)						0.621	**<0.001**	**<0.001**
♀ AT1-blockade	9.2 ± 3.3	13.4 ± 4.8	18.5 ± 4.7	23.1 ± 5.7	27.3 ± 7.1			
♀ PBO	8.4 ± 2.6	13.8 ± 3.5	18.7 ± 4.2	23.3 ± 5.2	27.5 ± 6.3			
♂ AT1-blockade	12.5 ± 4.4*	19.2 ± 5.9*	25.3 ± 6.3*	31.8 ± 7.5*	37.6 ± 9.4*			
♂ PBO	12.8 ± 4.8*	20.1 ± 5.6*	26.4 ± 7.2*	32.1 ± 7.4*	36.4 ± 8.2*			
a-vO_2_diff (mL O_2_ 100 mL)						0.325	**<0.001**	**<0.001**
♀ AT1-blockade	0.07 ± 0.03	0.09 ± 0.03	0.10 ± 0.02	0.12 ± 0.03	0.12 ± 0.03			
♀ PBO	0.07 ± 0.02	0.09 ± 0.02	0.10 ± 0.02	0.11 ± 0.02	0.13 ± 0.04			
♂ AT1-blockade	0.09 ± 0.03*	0.12 ± 0.03*	0.13 ± 0.03*	0.14 ± 0.03*	0.16 ± 0.02*			
♂ PBO	0.09 ± 0.03*	0.12 ± 0.02*	0.13 ± 0.02*	0.14 ± 0.03*	0.14 ± 0.03*			

Data are expressed as mean ± SD.

a Condition: AT1-blockade vs. PBO.

Significant *P* values (*P* < 0.05) for main factors in ANOVA (condition, sex, or exercise intensity) are highlighted in bold.

^†^ *P* < 0.05 between condition (AT1-blockade vs. PBO) in a given sex and exercise intensity.

*P < 0.05 between sexes (men vs. women) in a given condition and exercise intensity.

Interaction (condition × sex × exercise intensity) was observed for LV Q (*P* = 0.039).

### Effect of Angiotensin II Type 1 Receptor (AT1)-Blockade Before and After Exercise

**Figure 1.**
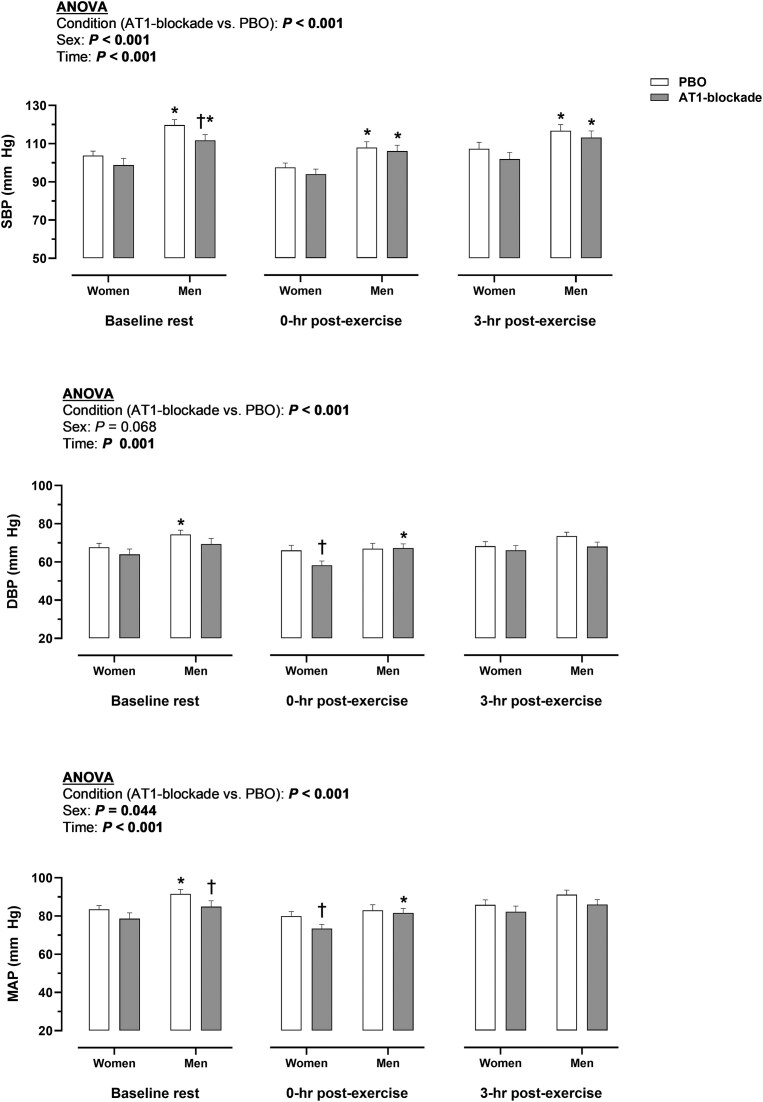
Effect of angiotensin II type 1 receptor (AT1)-blockade on arterial blood pressure before and after exercise. Data in bars represent mean values. Significant *P* values (*P* < 0.05) for main factors in ANOVA (condition, sex, or time) are highlighted in bold. ^†^*P* < 0.05 between condition (AT1-blockade vs. PBO) in a given sex and time point. *P < 0.05 between sexes (men vs. women) in a given condition and time point. Interactions (condition × sex × time or condition × time) were not observed (*P* ≥ 0.064).AT1-blockade, angiotensin II type 1 receptor blockade; DBP, diastolic arterial blood pressure; MAP, mean arterial blood pressure; PBO, placebo; SBP, systolic arterial blood pressure.

The effect of AT1-blockade on arterial blood pressure before and after exercise is illustrated in Figure 1. Considering together the resting time points of the study protocol (baseline rest, 0-h post-exercise, 3-h post-exercise), AT1-blockade decreased all arterial blood pressures (SBP, DBP, MAP) compared with PBO (*P* < 0.001). This effect was more acutely apparent for SBP in men and DBP in women. With respect to the effect of sex, men exhibited higher SBP and MAP than women during the resting time points (*P* ≤ 0.044). No interaction between condition, sex, and time was detected (*P* ≥ 0.064). The change in plasma volume from baseline rest to 3-h post-exercise did not differ between AT1-blockade and PBO conditions in men (−175±156 vs. −197±313 mL, *P* = 0.698) and women (−93±174 vs. −120±215 mL, *P* = 0.448).

**Figure 2.**
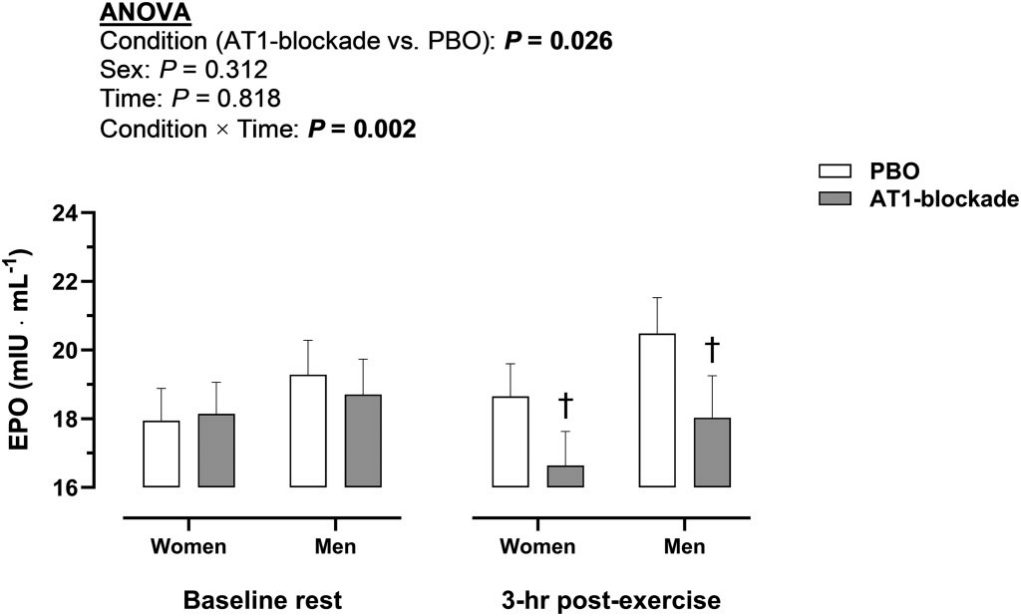
Effect of angiotensin II type 1 receptor (AT1)-blockade on circulating erythropoietin (EPO) before and after exercise. Data in bars represent mean values. Due to the ∼3-h time period required to observe exercise-induced changes in circulating EPO,^2^ post-exercise measurements were performed at the last time point (3-h post-exercise). Significant *P* values (*P* < 0.05) for main factors in ANOVA (condition, sex or time) are highlighted in bold. ^†^*P* < 0.05 between condition (AT1-blockade vs. PBO) in a given sex and time point. An interaction (condition × time) was observed for EPO (*P* = 0.002). AT1-blockade, angiotensin II type 1 receptor blockade; EPO, erythropoietin; PBO, placebo.

**Figure 3.**
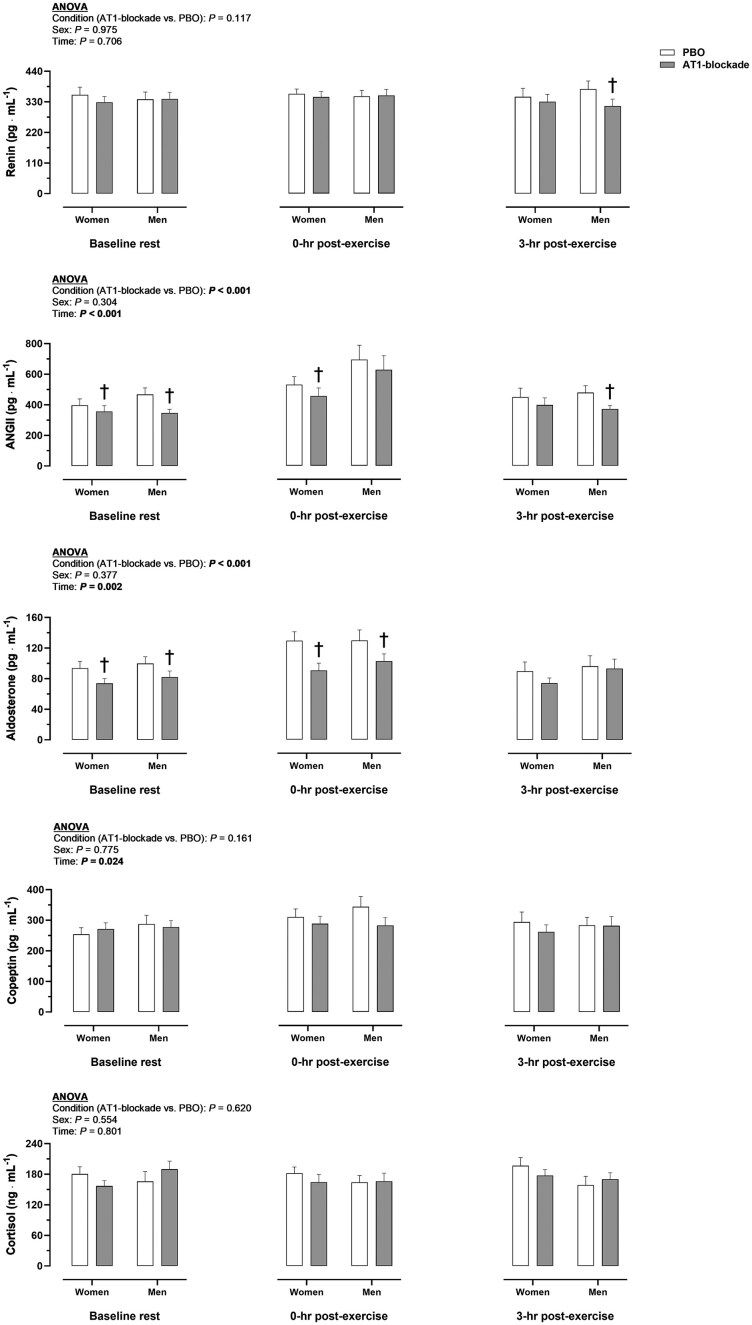
Effect of angiotensin II type 1 receptor (AT1)-blockade on erythropoietin (EPO)-regulating hormones before and after exercise. Data in bars represent mean values. Significant *P* values (*P* < 0.05) for main factors in ANOVA (condition, sex, or time) are highlighted in bold. ^†^*P* < 0.05 between condition (AT1-blockade vs. PBO) in a given sex and time point. Interactions (condition × sex × time or condition × time) were not observed (*P* ≥ 0.195). ANGII, angiotensin II; AT1-blockade, angiotensin II type 1 receptor blockade; PBO, placebo.

Figure 2 portrays the effect of AT1-blockade on circulating EPO before and after exercise. A condition (AT1-blockade vs. PBO, *P* = 0.026) and condition × time interaction (*P* = 0.002) was observed, such that AT1-blockade decreased EPO at 3-h post-exercise compared with PBO in women and men (*P* ≤ 0.025). No sex difference was observed in EPO in any condition and time point (*P* ≥ 0.199). Nonetheless, in the PBO condition, EPO was increased from rest to 3-h post-exercise in men (*P* = 0.041), whereas, in the AT1-blockade condition, EPO was reduced from rest to 3-h post exercise in women (*P* = 0.017). With respect to EPO-regulating hormones (Figure 3), ANGII and aldosterone were diminished with AT1-blockade vs. placebo (*P* < 0.001). ANGII, aldosterone and copeptin were generally increased at 0-h post-exercise (*P* ≤ 0.002), returning to baseline at 3-h post-exercise. Copeptin was also increased at 0-h post-exercise but only in the PBO condition (*P* ≤ 0.032). No sex effect or interaction was detected for EPO-regulating hormones (*P* ≥ 0.304).

## Discussion

This study determined whether a prevalently prescribed antihypertensive medication blocking the AT1 impairs the acute EPO response to exercise in healthy women and men. The main findings are: (1) AT1-blockade abolishes the acute EPO response to exercise irrespective of sex; (2) the exercise-induced increase in EPO-regulating hormones such as ANGII, aldosterone and copeptin is generally attenuated with AT1-blockade in women and men; (3) despite AT1-blockade slightly reduces left heart volumes and filling pressure during exercise, VO_2peak_ and whole-body O_2_ extraction are not affected by AT1-blockade in women and men.

EPO is a fairly stable hormone in the circulation, with no apparent circadian rhythm.^32^ Likewise, the acute EPO response to exercise is known to be small (+1-2 mIU mL^−1^), relative to the absolute and relative changes observed in other hormones.^2,3^ Approximately a 5-20% increment in circulating EPO is elicited by moderate- to high-intensity endurance exercise for a few hours, staying at this moderately higher level for the initial 4 wk of endurance training.^2^ In parallel, the total volume of circulating red blood cells (RBCV) and hemoglobin mass are proportionally augmented in 4-8 wk, which explain the improvement in aerobic capacity.^2,33^ In this regard, the expected increase of RBCV and hemoglobin mass elicited by 8 wk of endurance training is eliminated by daily administration of ACEi,^34^ known to reduce basal EPO by ∼20% in healthy adults.^10^ However, it is worth noticing that in order to pharmacologically elicit a 10% increment in hemoglobin mass in healthy adults, the minimum administered doses of recombinant human EPO (intravenously delivered) at least must double the erythropoietic activity (≈concentration) in blood of endogenous circulating EPO.^35^ Accordingly, the exercise-induced increment in EPO might not fully explain exercise training-induced erythropoiesis; additional hormones such as cortisol, catecholamines, growth hormone, and insulin-like growth factor 1 may contribute to erythropoiesis, coupled with the hyperplasia of the hematopoietic bone marrow.^3^ Nonetheless, increased EPO production in response to exercise is considered the fundamental exercise-induced endocrine signal to enhance erythropoiesis, until experimental evidence does not prove otherwise.^3^ In this study, the exercise-induced increment in EPO (+1 mIU mL^−1^) was negated and reverted following a moderate dose of valsartan (Figure 2), a common antihypertensive medication blocking AT1, denoting the importance of intact RAS, specifically ANGII signaling, for EPO production with exercise in healthy women and men.

The mechanisms underlying the EPO response to exercise have remained speculative since the early 1990s.^36^ Hematological, hemodynamic and endocrine factors influenced by exercise have been considered to contribute to the regulation of EPO.^3,37^ The latter two categories are commonly seen as intrinsically related: exercise leads to post-exercise peripheral vasodilation resulting in mild hypotension, which presumedly stimulates the production of hormones regulating blood pressure, EPO production and blood volume.^3,37,38^ In this regard, the exercise-induced increase in ANGII, aldosterone and copeptin was generally blunted with AT1-blockade (Figure 3), despite arterial blood pressures were further reduced in this condition (Figure 1). A potential explanation involves the fact that AT1-blockade necessarily enhances the activation of unblocked ANGII type 2 receptors (AT2),^39^ which increases the activity of angiotensin-converting enzyme 2 (ACE2), thus augmenting the hydrolysis of ANGII.^40^ The subsequent reduction in circulating ANGII, observed in this study with AT1-blockade (Figure 3), may explain, along with the direct effects of AT1-blockade on the adrenal glands and the supraoptic nucleus, the blunted aldosterone and copeptin responses to exercise.^41^ Whichever the mechanism, the endocrine, but not the primary hemodynamic, stimulation of EPO production was decreased concomitantly with the reduced EPO response to exercise. Accordingly, endocrine factors could be the ultimate regulators of the EPO response to exercise, being the mediators of the hemodynamic (hypotensive) effects on EPO production. In turn, the reduction in circulating EPO might per se lead to hypotension, as the relationship between blood pressure and EPO is reciprocal.^42^ The regulation of EPO with exercise is therefore intrinsically related to that of blood pressure but seems to be ultimately governed by interdependent feedback loops in the RAS and hypothalamic-pituitary-adrenal axis. Hence, the EPO response to exercise might be highly sensitive to pharmacologically- or lifestyle-related alterations in the endocrine system.

Regular exercise is typically recommended, as a cornerstone therapy, to antihypertensive patients.^43^ While the benefits of exercise on cardiovascular health are beyond discussion, its interaction with antihypertensive drugs altering the RAS is uncertain. Notably, AT1-blockade is expected to alter the hemodynamic determinants of aerobic capacity, possibly restraining key acute exercise (hemodynamic) stimuli and exercise intensities required to induce beneficial cardiovascular adaptations. In this respect, AT1-blockade resulted in smaller left heart volumes during incremental exercise, notably in men, leading to reduced cardiac pumping capacity (∼7% decrement) (Table 2). The potential mechanisms underlying the sex-specific reduction in LV Q during exercise in men with AT1-blockade are unclear. In both sexes, markedly in men, estimated LV filling pressure during exercise was lower with AT1-blockade (Table 2). Hence, AT1-blockade reduced the hemodynamic stress of the heart during exercise. This could be mainly explained by reduced cardiac (specifically: LV) preload and/or afterload. The latter can be discarded as arterial blood pressures and TPR during exercise were not affected by AT1-blockade (Table 2). On the other hand, reduced LV preload can be justified by the observed curtailment of LA volume, possibly leading to decreased LV passive and active filling. Whilst speculative, increased venous capacitance with AT1-blockade may decrease the venous gradient driving venous return and cardiac filling.^44^ Notwithstanding the aforementioned cardiac effects during exercise, whole-body O_2_ consumption (VO_2_) was not altered by AT1-blockade in women and men (Table 2). The fact that O_2_ extraction (a-vO2_diff_) was also unaltered by AT1-blockade denotes that efficient blood flow distribution in the arterial system was preserved, contrasting with the marked impairment of O_2_ extraction induced by powerful arterial dilator drugs during exercise.^45^ Collectively considered, AT1-blockade did alter central hemodynamics during exercise, but these alterations did not have a measurable impact on cardiac and aerobic capacities, which represent the strongest prognostic factors for mortality among exercise variables.^4,46^

Limitations. Given the pharmacological differences among antihypertensive medications that block the RAS, notably between ACEi and ARB, the present findings cannot be straightforwardly extrapolated to the whole class of these drugs.^6^ In addition, while the EPO response to exercise is deemed essential to enhance the number of circulating red blood cells and thereby aerobic capacity, additional hormones may contribute to exercise-induced erythropoiesis.^3^ Finally, the impact of long-term AT1-blockade on the effects of exercise training on erythropoiesis will have to be determined by longitudinal studies.

## Conclusions

Using a randomized, double-blind, and placebo-controlled design, this study assessed whether AT1-blockade, via commonly prescribed antihypertensive medication, inhibits EPO production with exercise—a key endocrine response to enhance erythropoiesis and aerobic capacity in humans. AT1-blockade eliminated the acute EPO response to exercise irrespective of sex. Moreover, the effect of exercise on EPO-regulating hormones was generally blunted with AT1-blockade in both sexes. Long-term placebo-controlled studies manipulating AT1 signaling via antihypertensive medications are needed to ascertain the functional and prognostic impact of chronic AT1-blockade on exercise training-induced adaptations in cardiac and aerobic capacities.

## Supplementary Material

zqaf032_Supplemental_File

## Data Availability

All data relevant to this study are presented in the manuscript and the supplementary material.
