# Supplementary material for: Blockade of Angiotensin II Receptor Type 1 Abolishes the Erythropoietin Response to Exercise
Source: Function (Oxf). 2025 Jul 21;6(4):zqaf032. doi: 10.1093/function/zqaf032 (PMC12620964; doi:10.1093/function/zqaf032)
Supplement: zqaf032_Supplemental_File [file zqaf032_supplemental_file.docx]

**SUPPLEMENTAL MATERIAL**

- **Supplemental** **Table 1**. Comprehensive data on the effect of angiotensin II type 1 receptor (AT1)-blockade on cardiac, hemodynamic, O_2_ consumption and extraction variables during incremental exercise.

**Supplemental Table 1**. Comprehensive data on the effect of angiotensin II type 1 receptor (AT1)-blockade on cardiac, hemodynamic, O_2_ consumption and extraction variables during incremental exercise

|  | **Exercise intensity (% HR_peak_)** | | | | | **Three-way ANOVA (*P* value)** | | |
| --- | --- | --- | --- | --- | --- | --- | --- | --- |
|  | **60** | **70** | **80** | **90** | **100** | **Condition^a^** | **Sex** | **Exercise intensity** |
| **Heart volumes & rate** | |  |  |  |  |  |  |  |
| RA (mL∙m^-2^) |  |  |  |  |  | 0.219 | 0.069 | **0.003** |
| ♀ AT1-blockade | 11.6 ± 2.1 | 11.7 ± 2.0 | 11.0 ± 2.3 | 11.0 ± 2.4 | 10.1 ± 2.2 |  |  |  |
| ♀ PBO | 11.6 ± 2.9 | 11.5 ± 2.8 | 11.2 ± 2.5 | 11.7 ± 2.5 | 11.4 ± 2.7 |  |  |  |
| ♂ AT1-blockade | 12.6 ± 3.4 | 12.3 ± 3.0 | 12.3 ± 3.7 | 12.6 ± 3.4 | 12.1 ± 3.6 |  |  |  |
| ♂ PBO | 12.7 ± 3.3 | 12.7 ± 3.0 | 12.4 ± 3.4 | 12.4 ± 3.5 | 12.3 ± 3.4 |  |  |  |
| LA (mL∙m^-2^) |  |  |  |  |  | **0.036** | 0.276 | **<0.001** |
| ♀ AT1-blockade | 12.8 ± 2.8 | 12.9 ± 2.3 | 12.2 ± 2.4 | 12.0 ± 2.7 | 11.3 ± 2.3† |  |  |  |
| ♀ PBO | 13.2 ± 3.3 | 12.8 ± 3.3 | 12.7 ± 2.9 | 12.9 ± 2.8 | 12.4 ± 2.6 |  |  |  |
| ♂ AT1-blockade | 13.3 ± 3.4 | 12.7 ± 3.2† | 13.1 ± 3.3 | 13.4 ± 3.8 | 12.8 ± 3.7 |  |  |  |
| ♂ PBO | 13.8 ± 3.8 | 13.6 ± 3.6 | 13.5 ± 3.8 | 13.3 ± 3.5 | 13.2 ± 3.3 |  |  |  |
| LVEDV (mL∙m^-2^) |  |  |  |  |  | **0.017** | **<0.001** | **0.001** |
| ♀ AT1-blockade | 61.7 ± 10.5 | 62.1 ± 10.4 | 63.8 ± 8.6 | 61.9 ± 9.5† | 64.7 ± 11.5 |  |  |  |
| ♀ PBO | 60.5 ± 10.5 | 62.7 ± 8.9 | 64.0 ± 9.5 | 65.0 ± 10.6 | 63.3 ± 10.3 |  |  |  |
| ♂ AT1-blockade | 72.4 ± 11.8* | 72.7 ± 11.4* | 72.2 ± 11.2†* | 74.2 ± 10.9* | 72.1 ± 10.0†* |  |  |  |
| ♂ PBO | 73.3 ± 12.2* | 74.3 ± 11.1* | 76.2 ± 10.9* | 76.9 ± 10.4* | 77.5 ± 12.0* |  |  |  |
| LVESV (mL∙m^-2^) |  |  |  |  |  | 0.729 | **<0.001** | **<0.001** |
| ♀ AT1-blockade | 11.2 ± 2.3 | 10.8 ± 2.6 | 10.6 ± 2.1 | 9.9 ± 2.3 | 9.9 ± 2.6 |  |  |  |
| ♀ PBO | 10.8 ± 2.0 | 10.7 ± 2.5 | 11.0 ± 2.5 | 10.1 ± 2.0 | 9.8 ± 1.6 |  |  |  |
| ♂ AT1-blockade | 14.1 ± 2.9* | 13.0 ± 2.2* | 13.3 ± 2.9* | 13.1 ± 3.0* | 12.1 ± 3.3* |  |  |  |
| ♂ PBO | 14.0 ± 3.3* | 13.1 ± 2.7* | 13.2 ± 2.6* | 12.5 ± 2.2* | 12.1 ± 2.2* |  |  |  |
| LV SV (mL∙m^-2^) |  |  |  |  |  | **0.005** | **<0.001** | **<0.001** |
| ♀ AT1-blockade | 50.5 ± 9.4 | 51.3 ± 9.1 | 53.1 ± 7.4 | 52.1 ± 8.1† | 54.9 ± 10.2 |  |  |  |
| ♀ PBO | 49.7 ± 9.0 | 52.0 ± 7.4 | 53.0 ± 7.8 | 54.8 ± 9.2 | 53.5 ± 9.6 |  |  |  |
| ♂ AT1-blockade | 58.3 ± 10.0* | 59.7 ± 10.0* | 58.9 ± 9.6†* | 61.1 ± 9.1†* | 60.0 ± 8.4†* |  |  |  |
| ♂ PBO | 59.3 ± 10.6* | 61.2 ± 10.2* | 63.0 ± 9.6* | 64.4 ± 9.0* | 65.4 ± 11.1* |  |  |  |
| HR (bpm) |  |  |  |  |  | 0.599 | 0.899 | **<0.001** |
| ♀ AT1-blockade | 91.9 ± 9.7 | 107.5 ± 13.0 | 122.5 ± 15.2 | 136.8 ± 15.8 | 151.3 ± 17.8 |  |  |  |
| ♀ PBO | 91.1 ± 10.3 | 106.2 ± 12.0 | 121.9 ± 13.5 | 136.9 ± 15.2 | 150.7 ± 17.3 |  |  |  |
| ♂ AT1-blockade | 91.4 ± 14.1 | 107.0 ± 15.5 | 122.7 ± 17.8 | 138.8 ± 20.5 | 151.8 ± 22.0 |  |  |  |
| ♂ PBO | 91.8 ± 13.7 | 107.3 ± 14.4 | 123.5 ± 17.6 | 138.1 ± 19.9 | 149.5 ± 22.2 |  |  |  |
| LV Q (L∙min^-1^∙m^-2^) |  |  |  |  |  | **0.029** | **0.002** | **<0.001** |
| ♀ AT1-blockade | 4.64 ± 1.03 | 5.51 ± 1.16 | 6.49 ± 1.14 | 7.13 ± 1.39 | 8.31 ± 1.90 |  |  |  |
| ♀ PBO | 4.55 ± 1.06 | 5.52 ± 0.99 | 6.46 ± 1.13 | 7.52 ± 1.57 | 8.06 ± 1.72 |  |  |  |
| ♂ AT1-blockade | 5.35 ± 1.32* | 6.39 ± 1.46* | 7.22 ± 1.54* | 8.44 ± 1.62* | 9.09 ± 1.79 |  |  |  |
| ♂ PBO | 5.42 ± 1.18* | 6.56 ± 1.38* | 7.76 ± 1.53* | 8.87 ± 1.71* | 9.75 ± 2.08* |  |  |  |
| **LV function** | |  |  |  |  |  |  |  |
| Mitral E/A |  |  |  |  |  | 0.190 | 0.279 | **0.010** |
| ♀ AT1-blockade | 1.10 ± 0.25 | 1.09 ± 0.18 | 1.10± 0.21 | 1.13 ± 0.20 | 1.17 ± 0.16 |  |  |  |
| ♀ PBO | 1.16 ± 0.30 | 1.12 ± 0.22 | 1.12 ± 0.18 | 1.09 ± 0.19 | 1.21 ± 0.29 |  |  |  |
| ♂ AT1-blockade | 1.12 ± 0.27 | 1.12 ± 0.36 | 1.11 ± 0.30 | 1.15 ± 0.31 | 1.22 ± 0.32 |  |  |  |
| ♂ PBO | 1.11 ± 0.29 | 1.14 ± 0.30 | 1.19 ± 0.24 | 1.24 ± 0.24 | 1.25 ± 0.35 |  |  |  |
| Myocardial e’ (cm·s^-1^) | |  |  |  |  | 0.059 | 0.914 | **<0.001** |
| ♀ AT1-blockade | 17.8 ± 2.7 | 19.1 ± 2.6 | 19.8 ± 3.6 | 20.2 ± 3.3 | 21.4 ± 4.4 |  |  |  |
| ♀ PBO | 16.5 ± 2.6 | 17.8 ± 2.6 | 19.5 ± 2.6 | 19.9 ± 3.5 | 21.3 ± 3.9 |  |  |  |
| ♂ AT1-blockade | 17.4 ± 2.8 | 18.7 ± 2.9 | 19.5 ± 2.6 | 20.2 ± 3.4 | 21.5 ± 4.6 |  |  |  |
| ♂ PBO | 17.2 ± 3.5 | 18.5 ± 3.2 | 19.2 ± 2.7 | 20.1 ± 3.3 | 21.4 ± 4.1 |  |  |  |
| Myocardial a’ (cm·s^-1^) | |  |  |  |  | 0.577 | 0.729 | **<0.001** |
| ♀ AT1-blockade | 16.8 ± 3.5 | 18.8 ± 4.1 | 19.3 ± 4.1 | 19.5 ± 4.3 | 20.8 ± 4.3 |  |  |  |
| ♀ PBO | 15.6 ± 2.9 | 17.3 ± 3.9 | 19.3 ± 3.6 | 19.5 ± 3.9 | 21.0 ± 4.4 |  |  |  |
| ♂ AT1-blockade | 16.1 ± 3.7 | 18.0 ± 3.4 | 19.6 ± 4.9 | 21.3 ± 4.7 | 19.6 ± 4.7 |  |  |  |
| ♂ PBO | 16.7 ± 3.0 | 18.9 ± 3.8 | 19.8 ± 4.8 | 19.6 ± 4.8 | 20.3 ± 5.6 |  |  |  |
| LV E/e’ |  |  |  |  |  | **0.006** | 0.114 | **<0.001** |
| ♀ AT1-blockade | 5.19 ± 1.15 | 5.42 ± 1.03 | 5.98 ± 1.99 | 5.98 ± 1.27 | 6.05 ± 1.43 |  |  |  |
| ♀ PBO | 5.58 ± 1.35 | 5.95 ± 1.33 | 5.87 ± 1.26 | 6.15 ± 1.66 | 6.10 ± 1.39 |  |  |  |
| ♂ AT1-blockade | 4.91 ± 0.88 | 4.96 ± 1.21† | 5.08 ± 1.47† | 5.46 ± 1.99 | 5.59 ± 1.65 |  |  |  |
| ♂ PBO | 5.27 ± 1.21 | 5.57 ± 1.29 | 5.92 ± 1.27 | 6.21 ± 1.46 | 6.15 ± 1.40 |  |  |  |
| **LV work, arterial blood pressure & vascular resistance** | | | |  |  |  |  |  |
| LV work (mm Hg·L·min^-1^) | |  |  |  |  | 0.054 | **<0.001** | **<0.001** |
| ♀ AT1-blockade | 663 ± 202 | 826 ± 241 | 1020 ± 277 | 1190 ± 379 | 1452 ± 573 |  |  |  |
| ♀ PBO | 665 ± 173 | 842 ± 223 | 1080 ± 298 | 1322 ± 368 | 1461 ± 465 |  |  |  |
| ♂ AT1-blockade | 932 ± 327* | 1183 ± 405* | 1394 ± 501* | 1738 ± 566* | 1906 ± 526* |  |  |  |
| ♂ PBO | 969 ± 312* | 1239 ± 404* | 1560 ± 487* | 1884 ± 564* | 2112 ± 677* |  |  |  |
| SBP (mm Hg) |  |  |  |  |  | 0.835 | **0.029** | **<0.001** |
| ♀ AT1-blockade | 116.2 ± 20.3 | 116.2 ± 22.1 | 121.6 ± 23.4 | 127.1 ± 27.2 | 128.7 ± 32.8 |  |  |  |
| ♀ PBO | 115.6 ± 22.9 | 118.1 ± 26.3 | 124.4 ± 27.8 | 129.0 ± 26.0 | 131.0 ± 27.9 |  |  |  |
| ♂ AT1-blockade | 129.2 ± 19.0* | 133.2 ± 22.0* | 135.6 ± 27.2 | 142.9 ± 29.1* | 143.1 ± 29.1 |  |  |  |
| ♂ PBO | 127.5 ± 22.9* | 130.2 ± 25.6* | 133.2 ± 27.8 | 138.7 ± 33.1 | 139.0 ± 34.0 |  |  |  |
| DBP (mm Hg) |  |  |  |  |  | 0.137 | 0.236 | **<0.001** |
| ♀ AT1-blockade | 75.9 ± 15.2 | 80.5 ± 18.5 | 86.4 ± 22.0 | 93.1 ± 24.3 | 97.8 ± 29.9 |  |  |  |
| ♀ PBO | 78.0 ± 13.7 | 81.5 ± 16.1 | 90.4 ± 19.8 | 98.5 ± 21.2 | 103.2 ± 21.9 |  |  |  |
| ♂ AT1-blockade | 78.9 ± 17.6 | 85.1 ± 20.5 | 91.1 ± 24.4 | 99.4 ± 25.8 | 105.3 ± 24.6 |  |  |  |
| ♂ PBO | 81.9 ± 15.4 | 88.5 ± 15.7 | 96.3 ± 18.7 | 104.7 ± 22.1 | 108.2 ± 24.1 |  |  |  |
| MAP (mm Hg) |  |  |  |  |  | 0.168 | 0.282 | **<0.001** |
| ♀ AT1-blockade | 92.9 ± 17.6 | 96.3 ± 19.6 | 101.9 ± 21.9 | 108.1 ± 24.6 | 111.0 ± 30.0 |  |  |  |
| ♀ PBO | 95.5 ± 17.0 | 98.9 ± 19.9 | 106.4 ± 22.0 | 112.9 ± 21.6 | 116.3 ± 22.4 |  |  |  |
| ♂ AT1-blockade | 96.7 ± 17.8 | 102.2 ± 19.7 | 107.0 ± 23.6 | 114.6 ± 24.3 | 118.4 ± 23.1 |  |  |  |
| ♂ PBO | 99.2 ± 16.0 | 104.8 ± 17.1 | 110.9 ± 20.1 | 118.2 ± 24.4 | 120.3 ± 26.0 |  |  |  |
| TPR (dyn·s·cm^-5^) |  |  |  |  |  | 0.726 | **<0.001** | **<0.001** |
| ♀ AT1-blockade | 1088 ± 2.92 | 937 ± 2.72 | 836 ± 223 | 816 ± 222 | 712 ± 201 |  |  |  |
| ♀ PBO | 1150 ± 331 | 957 ± 234 | 866 ± 224 | 808 ± 248 | 775 ± 194 |  |  |  |
| ♂ AT1-blockade | 851 ± 234* | 745 ± 218* | 691 ± 197* | 627 ± 163* | 614 ± 190 |  |  |  |
| ♂ PBO | 854 ± 212* | 741 ± 156* | 654 ± 136* | 614 ± 155* | 573 ± 155* |  |  |  |
| **Whole-body O_2_ uptake and extraction** | | |  |  |  |  |  |  |
| VO_2_ (mL∙min^-1^∙kg^-1^) |  |  |  |  |  | 0.621 | **<0.001** | **<0.001** |
| ♀ AT1-blockade | 9.2 ± 3.3 | 13.4 ± 4.8 | 18.5 ± 4.7 | 23.1 ± 5.7 | 27.3 ± 7.1 |  |  |  |
| ♀ PBO | 8.4 ± 2.6 | 13.8 ± 3.5 | 18.7 ± 4.2 | 23.3 ± 5.2 | 27.5 ± 6.3 |  |  |  |
| ♂ AT1-blockade | 12.5 ± 4.4* | 19.2 ± 5.9* | 25.3 ± 6.3* | 31.8 ± 7.5* | 37.6 ± 9.4* |  |  |  |
| ♂ PBO | 12.8 ± 4.8* | 20.1 ± 5.6* | 26.4 ± 7.2* | 32.1 ± 7.4* | 36.4 ± 8.2* |  |  |  |
| a-vO_2_diff (mL O_2_· 100 mL) | |  |  |  |  | 0.325 | **<0.001** | **<0.001** |
| ♀ AT1-blockade | 0.07 ± 0.03 | 0.09 ± 0.03 | 0.10 ± 0.02 | 0.12 ± 0.03 | 0.12 ± 0.03 |  |  |  |
| ♀ PBO | 0.07 ± 0.02 | 0.09 ± 0.02 | 0.10 ± 0.02 | 0.11 ± 0.02 | 0.13 ± 0.04 |  |  |  |
| ♂ AT1-blockade | 0.09 ± 0.03* | 0.12 ± 0.03* | 0.13 ± 0.03* | 0.14 ± 0.03* | 0.16 ± 0.02* |  |  |  |
| ♂ PBO | 0.09 ± 0.03* | 0.12 ± 0.02* | 0.13 ± 0.02* | 0.14 ± 0.03* | 0.14 ± 0.03* |  |  |  |

Data are expressed as mean ± SD.

^a^ Condition: AT1-blockade vs. PBO.

Significant *P* values (*P* < 0.05) for main factors in ANOVA (condition, sex or exercise intensity) are highlighted in bold.

† *P* < 0.05 between condition (AT1-blockade vs. PBO) in a given sex and exercise intensity.

* *P* < 0.05 between sexes (men vs. women) in a given condition and exercise intensity.

Interaction (condition × sex × exercise intensity) was observed for LV Q (*P*=0.039).

AT1-blockade, angiotensin II type 1 receptor blockade; a-vO_2_diff, arteriovenous O_2_ difference; DBP, diastolic arterial blood pressure; HR, heart rate; HR_peak_, peak heart rate; LA, left atrial volume; LV, left ventricle; LV E/e’, ratio of peak blood flow velocity to tissue myocardial velocity at early diastole in the left ventricle; LVEDV, left ventricular end-diastolic volume; LVESV, left ventricular end-systolic volume; LV Q, left ventricular cardiac output; LV SV, left ventricular stroke volume; MAP, mean arterial blood pressure; Mitral E/A, ratio of peak blood flow velocity in early diastole (E wave) to peak blood flow velocity in late diastole due to atrial contraction (A wave); Myocardial a’, tissue myocardial velocity at late diastole; Myocardial e’, tissue myocardial velocity at early diastole; PBO, placebo; RA, right atrial volume; SBP, systolic arterial blood pressure; TPR, total peripheral resistance to blood flow; VO_2_, pulmonary O_2_ uptake.
